# Acupuncture for the Treatment of Opiate Addiction

**DOI:** 10.1155/2012/739045

**Published:** 2012-02-22

**Authors:** Jaung-Geng Lin, Yuan-Yu Chan, Yi-Hung Chen

**Affiliations:** ^1^School of Chinese Medicine, China Medical University, No. 91 Hsueh-Shih Road, Taichung 40402, Taiwan; ^2^Graduate Institute of Integrated Medicine, China Medical University, No. 91 Hsueh-Shih Road, Taichung 40402, Taiwan; ^3^Department of Psychiatry, Armed Forces Taoyuan General Hospital, No. 168 Zhong-Xing Road, Taoyuan 32551, Taiwan; ^4^Graduate Institute of Acupuncture Science, China Medical University, No. 91 Hsueh-Shih Road, Taichung 40402, Taiwan

## Abstract

Acupuncture is an accepted treatment worldwide for various clinical conditions, and the effects of acupuncture on opiate addiction have been investigated in many clinical trials. The present review systematically analyzed data from randomized clinical trials published in Chinese and English since 1970. We found that the majority agreed on the efficacy of acupuncture as a strategy for the treatment of opiate addiction. However, some of the methods in several included trials have been criticized for their poor quality. This review summarizes the quality of the study design, the types of acupuncture applied, the commonly selected acupoints or sites of the body, the effectiveness of the treatment, and the possible mechanism underlying the effectiveness of acupuncture in these trials.

## 1. Introduction

Acupuncture, the practice of inserting thin solid needles into specific documented points of the body to treat many different disorders, has been practiced in China since 2500 BC [1]. Acupuncture is gaining popularity in Western countries as an alternative and complementary therapeutic intervention, and this therapeutic technique is also growing in popularity worldwide [2, 3]. Acupuncture is based on the principles of traditional oriental medicine and was developed according to the principle that human bodily functions are controlled by the “meridian” and “Qi and blood” systems. There are 365 designated acupuncture points located along these meridians that can be used for stimulation through needles to balance and harmonize the yin and yang by relieving blockages in the flow of Qi [4]. This method of healing has been used to promote balance in and improve the functions of the body's organs.

Acupuncture needles are either manipulated manually or via an electrical stimulator, that is, “electroacupuncture” (EA). New methods for stimulating the acupoints include applying electric current to skin electrodes over the points, directing a laser light onto the points, or using finger pressure to massage selected points (acupressure). In addition, many new points and entire “microsystems” of points have been described for specific body parts, for example, scalp acupuncture and ear acupuncture (auricular acupuncture). Acupuncture may be useful as an adjunct treatment in comprehensive management programs and might be efficacious in the treatment of pain [5] such as postoperative pain [6], benign prostate hyperplasia [7], nausea due to pregnancy, and postoperative and chemotherapy-induced nausea and vomiting [4]. Scalp acupuncture therapy appears to improve neurological deficits in patients with acute intracerebral hemorrhage [8]. Modern research is confirming the efficacy of auricular acupuncture for analgesia and anxiety-related diseases [9].

Acupuncture or EA stimulation typically elicits a composite of sensations termed “DeQi,” manifesting as soreness, numbness, heaviness, and distention, which are believed to reflect the efficacy of the treatment [10].

In 1996, the World Health Organization (WHO) listed 64 medical problems that were considered suitable for acupuncture treatment, including the treatment of drug abuse. There are 3 major advantages regarding the use of acupuncture to treat drug addiction. First, acupuncture therapy for opiate addiction is inexpensive, simple and has no side effects [11]. Second, acupuncture can be used for the prevention of opiate relapse [12]. Third, acupuncture therapy is safe for pregnant and parturient women [13].

The application of acupuncture to opiate addiction originated from a serendipitous observation by Dr. Wen in Hong Kong in 1972. Dr. Wen reported that acupuncture combined with electrical stimulation at 4 body points and 2 ear points relieved the symptoms of opioid withdrawal in persons with opiate addiction [14].

This method was later adopted in many clinical settings in Western countries, using a protocol developed in 1985 by the head of the US National Acupuncture Detoxification Association (NADA), Dr. M. Smith. The NADA protocol describes the insertion of 5 needles without the use of electrical stimulation bilaterally into the outer ear or auricle at points termed sympathetic, shenmen, kidney, lung, and liver. The NADA protocol advises that 5-point auricular acupuncture relieves withdrawal symptoms, prevents symptoms of craving, and increases patient participation rates in long-term treatment programs [15].

Auricular acupuncture is the most common form of acupuncture treatment for substance addiction in both the USA and the UK [16, 17]. In both countries, there are currently over 250 hospitals practicing acupuncture based on the NADA protocol [11].

A recent advance in this field was made by Dr. Han of Beijing's Peking University, whose 2005 protocol describes the placement of self-sticking electrodes to the skin over the acupoint followed by electrical stimulation to ameliorate opiate withdrawal signs and prevent relapse of heroin use. The device used for this purpose was named Han's acupoint nerve stimulator (HANS) [14].

### 1.1. Possible Mechanisms for the Effectiveness of Acupuncture on Opiate Addiction

The mesolimbic dopamine system originates in the ventral tegmental area (VTA) and projects to regions that include the nucleus accumbens and prefrontal cortex, which are believed to play a pivotal role in the development of opiate addiction [4]. Opiate abuse-induced changes in the levels of dopamine in the brain are associated with feelings of well-being and pleasure, providing positive reinforcement of continued opiate abuse [18, 19]. Conversely, withdrawal from chronic opiate administration reduces dopamine outflow in the nucleus accumbens [20, 21]. Opioid withdrawal causes dysphoria and significant distress, a state that addicts seek to avoid and one that can be a major motive for continuing opiate use (i.e., negative reinforcement) [22, 23].

Many studies in animals and humans have demonstrated that acupuncture causes multiple biological responses [24]. Manual acupuncture (MA) and EA are capable of triggering a chain of events that can be understood through controlled experiments. The best-known mechanism is via endogenous opiates and their receptors. Different kinds of endogenous opiates, such as  *β*-endorphin, enkephalin, endomorphin, and dynorphin, reportedly act as frequency-dependent factors in EA. EA of low frequency (2 Hz) accelerated the release of *β*-endorphin and enkephalin in the CNS whereas EA of high frequency (100 Hz) accelerated the release of dynorphin [25–28].

Early works have demonstrated the involvement of  *κ*  opioid receptors in the mechanism underlying the effects of acupuncture on morphine addiction. In 1993, Han and Zhang reported the effectiveness of EA on morphine abstinence syndrome in a rat experimental model. The authors found that 100 Hz EA produced a statistically significant suppression of wet shakes, teeth chattering, escape attempts, weight loss, and penile licking (*P* < 0.05) whereas 2 Hz EA produced a mild but significant suppression in escape attempts and wet shakes [29]. These results suggest that 100 Hz EA was far more effective than 2 Hz EA in suppressing withdrawal syndrome. Further studies suggested that EA suppresses opiate withdrawal syndrome by activating  *κ*  opioid receptors and dynorphin release [29–33].

Additionally, acupuncture affects the reinforcing effects of morphine. The method of conditioned place preference (CPP) is a commonly used animal model of drug craving [34]. Wang et al. reported that morphine-induced place preference in rats is significantly suppressed by 2 Hz EA and 2/100 Hz, but not at 100 Hz [35]. However, Shi et al. showed that 100 Hz EA significantly attenuated morphine-induced CPP, and this effect was completely blocked by *δ*- and  *κ*-opioid receptor antagonists, suggesting that the anticraving effects induced by 100 Hz EA are mediated by the activation of  *δ*- and  *κ*-opioid receptors [36].

In 2008, Yang et al. reviewed the possible mechanism underlying the effectiveness of acupuncture in the treatment of drug addiction and this review provided clear evidence for the biological effects underlying the use of acupuncture to treat drug abuse [4]. This review provided a hypothetical model of the effects of acupuncture on dopamine release in the nucleus accumbens. Regarding positive reinforcement, acupuncture treatment activates GABA_B_ receptors on the dopamine cell body and activates presynaptic  *κ*-opioid receptors in the nucleus accumbens through dynorphin neurons, resulting in decreased dopamine release. Regarding negative reinforcement, acupuncture treatment stimulates enkephalin neurons in the hypothalamus and interacts with  *μ*-opioid receptors to inhibit VTA GABAergic interneurons and thus increases dopamine release in the nucleus accumbens [4].

Recent basic studies further support the above-mentioned theory and additionally suggest a role for brain-derived neurotrophic factor (BDNF) in this process. MA at Shenmen (HT7) points regulates the reinforcing effects of morphine via regulation of GABA receptors [37] and significantly suppresses morphine-induced increases in locomotor activity and Fos expression in the nucleus accumbens and striatum [38]. Further, results from several animal studies [39–41] showed that both 2 and 100 Hz EA facilitate the recovery of VTA dopaminergic neurons damaged by chronic morphine administration and upregulate BDNF protein levels in the VTA, suggesting that the potential use of EA as a therapy for treating opiate addiction is associated with the activation of endogenous BDNF.

In summary, neurochemical and behavioral evidence have shown that acupuncture helps reduce the effects of positive and negative reinforcement involved in opiate addiction by modulating mesolimbic dopamine neurons. Moreover, several brain neurotransmitter systems involving opioids and GABA have been implicated in the modulation of dopamine release by acupuncture. However, many unanswered questions remain regarding the basic mechanisms of action of acupuncture. Future research could better determine the influence of acupuncture therapy on the regulation of dopamine and other neurotransmitters.

This paper provides an overview of trials that have investigated the clinical effectiveness of acupuncture in the treatment of opiate addiction. We here summarize the quality of the study design, types of acupuncture applied, commonly selected acupoints or sites of the body, and the effectiveness of the treatment in these trials.

## 2. Methods

### 2.1. Literature Search

In April 2011, a literature search was performed using the following English language databases: PubMed and EBSCOhost. The first search keyword used was “acupuncture” and the second keyword used was either “heroin” or “opiate”.

### 2.2. Inclusion and Exclusion Criteria

We included studies that met the following criteria: (1) randomized control trials (RCTs) that adopted a double-blind, single-blind, or nonblind design and (2) participants met criteria for opiate/heroin dependence.

Exclusion criteria included (1) nonnumeric data, (2) comments and replies, and (3) animal study.

### 2.3. Data Extraction and Quality Assessment

Clinical trials on the treatment of opiate/heroin dependence were selected based on the predetermined inclusion and exclusion criteria. Data were extracted from study reports by one reviewer and were verified by a second reviewer. The following key information was extracted from each study: first author, publication year, study design, sample size, characteristics of participants, main acupoints/sites selected, outcome measures, results reported, and adverse events.

We assessed the quality of the studies using the Jadad scale [42], which rates studies for (1) randomization, (2) double blinding, (3) description of withdrawal, (4) description of randomization, and (5) description of blinding. Trials scoring 1 or 2 points were considered of low quality whereas trials scoring 3–5 points were considered of high quality.

## 3. Results

**Figure 1:**
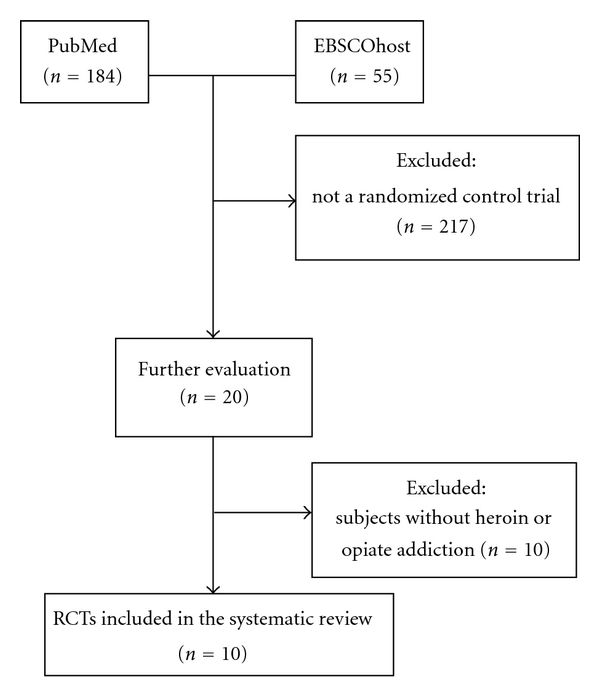
Flow diagram showing the number of studies included and excluded from the systematic review.

An initial search identified 184 published articles from PubMed and 55 published articles from EBSCOhost. Only 10 published articles met our inclusion criteria and these were systematically reviewed (Table 1). The other articles were excluded because they were not RCTs or included subjects without heroin or opiate addiction (Figure 1).

### 3.1. Types of Studies

Five studies mentioned the process of randomization. None of the studies mentioned the use of blinding on clinicians, subjects, or the raters of study outcomes. Four studies [45, 47, 48, 50, 51] were from Chinese journals.

### 3.2. Diagnostic Criteria and Characteristics of Participants

Five studies [47, 48, 50, 52] used the Diagnostic and Statistical Manual of Mental Disorders (DSM III, III-R, IV) criteria on opiate dependence, 1 study [47] used the Chinese Classification of Mental Disorders (CCMD II-R), 1 study [51] used the International Statistical Classification of Diseases and Related Health Problems (ICD-10) criteria on opiate dependence, and 4 studies [43, 44, 46, 49] did not mention the criteria used in diagnosing opiate dependence.

Ten studies involving 1034 subjects (including those in intervention groups and in control groups) were enrolled, of which 711 cases were from China, 200 were from the USA, 83 were from the UK, and 40 were from Iran. Forty participants were HIV positive.

### 3.3. Type of Intervention and Needling Method

Four studies [43, 44, 49, 52] used auricular acupuncture, 4 studies [46–48, 50] used body acupuncture with manual stimulation, 1 study [51] used body acupuncture with electrical stimulation, and 1 study [45] used HANS on the treatment group.

The reported courses of treatment in 6 studies [45–48, 50, 52] were between 1 to 2 weeks. The courses of the remaining studies were 3 days, 3 weeks, 8 weeks, and 6 months.

### 3.4. Outcome Measures and Effectiveness Assessment

In most of the reviewed studies, the outcome measures included attendance rate, craving scale, and opiate withdrawal symptoms. Seven studies [43, 45–48, 50, 51] provided evidence for acupuncture as treatment for opiate addiction whereas 3 studies [44, 49, 52] were against the use of acupuncture to treat opiate addiction.

### 3.5. Adverse Effects or Events

Most studies did not mention adverse effects or events, few studies described the monitoring of safety, and only 2 studies [43, 47] reported adverse events including slight bleeding, mild nausea, dizziness, and mild dry mouth.

### 3.6. Main Acupoints/Sites Selected

In the studies included in the review, several used a fixed set of acupoints or sites on their subjects and 1 study allowed some flexibility and needled additional points based on the symptom presentation of individual subjects. The 5 ear acupoints (sympathetic, shenmen, kidney, lung, and liver) were often used in the USA and UK. In China, the acupoints of Zusanli (ST36), Sanyinjiao (SP6), Hegu (LI4), and Neiguan (PC6) were most frequently used for the treatment of opiate addiction. A summary of the main acupoints or sites selected in the studies is presented in Table 2.

### 3.7. Methodological Quality

Eight of the 10 trials [43, 45–51] reviewed in this paper were classified as having low quality according to Jadad's methodological quality assessment [42], scoring 2 or fewer points. The remaining 2 studies [44, 52] scored >2 points and were classified as having higher quality. All methodological quality scores are presented in Table 3.

## 4. Discussion

Although many studies have reported positive findings regarding the use of acupuncture to treat drug dependence, the evidence for its effectiveness has been inconclusive and difficult to interpret [53]. There are few randomized controlled clinical trials of acupuncture treatment for opiate addiction, and the methodological methods used in several clinical trials of acupuncture treatment for opiate dependence can be criticized for their poor quality. The quality issues include the following: small numbers of patients, no control subjects, lack of randomized assignment, lack of details regarding specific point locations for needle insertion, and no specification regarding the degree of blinding among research subjects.

In this paper, we classified trials as having low quality if they lacked double-blinding, description of withdrawal, and description of randomization. The majority of low-scoring trials displayed positive results regarding acupuncture treatment for opiate addiction. Further, acupuncture treatment showed potential for preventing relapse and reducing the severity of withdrawal symptoms.

Studies receiving a high methodological quality score produced interesting results. Two studies [44, 52] received high methodological quality scores but failed to report auricular acupuncture effectively. These 2 studies produced negative results, reporting that auricular acupuncture had no effect on withdrawal severity, craving, and attendance when provided as an adjunct to methadone treatment services.

Four studies [43, 44, 49, 52] used auricular acupuncture for the treatment of heroin addiction and 3 of these studies [44, 49, 52] did not report any clinical gains from acupuncture for the treatment of heroin addiction. Five studies [46–48, 50, 51] used body acupuncture with manual or electrical stimulation and all reported some clinical efficacy from the acupuncture for the treatment of heroin addiction. The single study that used HANS [45] for the treatment of heroin addiction reported a significant improvement in the severity of withdrawal syndrome.

Although most of the articles from China reviewed herein have favorable outcome, the type of intervention and needling methods were different between the studies from China and Western countries. Most studies from China used body acupuncture to treat opiate addiction whereas studies from the other countries used auricular acupuncture to treat opiate addiction. In addition, there are various differences in the auricular acupuncture system in different countries. These findings are intriguing considering that these body and auricular points exhibited different efficacies regarding the use of acupuncture to treat opiate addiction.

The most frequently used points or sites for the treatment of opiate addiction by acupuncturists are grouped below based on their locations: points on the extremities: Zusanli (ST36), Sanyinjiao (SP6), Hegu (LI4), and Neiguan (PC6); points and areas on the trunk: Jiaji (EX-B2), Shenshu (BL23), Sishencong (EX-HN1), Baihui (GV20), and Dazhui (GV14); and points on the ear: sympathetic, shenmen, kidney, and lung.

Adverse events associated with acupuncture are infrequently reported and only 2 studies reviewed herein [43, 47] reported adverse events. Ernst and White [54] determined the range of incidence of adverse events associated with acupuncture and found that those most commonly reported were needle pain (1–45%), tiredness (2–41%), and bleeding (0.03–38%), whereas fainting and syncope were uncommon (0–0.3%), pneumothorax was rare, but feelings of relaxation were very common (86%).

Acupuncture is based on the complex TCM theory that an energy (Qi) flows through meridians in each organ and most acupoints are located along one of these meridians. Because diseases are caused by an imbalance or disturbance of Qi, needling at these acupoints can harmonize Qi and cure diseases. Our experience suggests that better therapeutic acupuncture effects are obtained by doctors with several years, or even decades, of clinical training. Without sufficiently trained practitioners, specific therapeutic results may be masked by nonspecific and even placebo effects. Most modern acupuncture trials provide qualification details of the practitioners that performed the therapies. In several trials [44, 52], details of the practitioners' training were merely acceptable whereas other trials did not provide this information. Therefore, the results and conclusions of these trials do not totally represent clinical settings.

The weakness of this review is the lack of available high-quality data and the results should be interpreted with caution because of the lack of well-designed, high-quality randomized controlled studies. Many studies did not use standard treatment protocols, objective diagnostic criteria, standardized outcome measures, and effective assessment methods. The methodological quality and the description of the studies were poor in the majority of studies.

It is appropriate for a systematic review to calculate the results of each study identified by the study authors only when those studies are sufficiently comparable as to subjects, interventions, and outcomes, and similar enough in design. In addition, the effects of a study intervention on the consequent health or outcomes have to lie in the same direction or show homogeneity. Under these conditions, the individual estimates from each study can be combined to produce a pooled estimate of effect, which is usually more precise than the evidence provided by any of the individual studies. When these conditions cannot be met, it is difficult to interpret the combined findings from individual studies consisting of heterogeneous subjects, interventions, and outcomes.

Although the 10 studies identified by our systematic review shared the same design (randomized control trial), they differed in their inclusion criteria, mode of intervention, and outcome measures. In particular, although 5 outcome measures were used by more than one study (i.e., attendance rate, retention rate, urinalysis, cravings, and withdrawal symptoms), the operational definitions for these measures differed by duration and units of measure. This study heterogeneity prevented us from conducting a statistical analysis.

## 5. Conclusion

This review covered a wide body of Chinese and English research investigations into the use of acupuncture for the treatment of opiate dependence from the early 1970s up to 2011. After 35 years of active research by both Asian and Western scientists, this review cannot be used to establish the efficacy of acupuncture in the treatment of opiate addiction because the majority of these studies were classified as having low quality. Although this review may provide a basis for clinicians and future research, future well-designed RCT studies are needed to confirm the efficacy of acupuncture in the treatment of opiate addiction.

## Figures and Tables

**Table 1:** Summary of studies included in the review.

Author (year)	Jadad score	No. of subjects (acup/control)	Mean age (male%)	Inclusion criteria	Intervention type	Treatment frequency (duration)	Treated acupoints	Type of control group	Outcome measure	Results reported	Adverse events
Washburn et al. (1993), [43] USA	1	100 (55/45)	(1) Standard treatment group: 40.5 (63%) (2) Sham treatment group: 40.4 (73%)	Self-reported with history of intravenous use of heroin confirmed by physical examination for signs of recent needle use	AA	Daily (21 days)	Sympathetic Shenmen Kidney Lung	Nonspecific points	(1) Attendance rate (2) Self reports of frequency of heroin use (3) Urinalysis	(1) *P* < 0.05 (2) *P* < 0.01 (3) NS	(1) Slight bleeding (2) Mild nausea (3) Mild dizziness

Wells et al. (1995), [44] USA	3	60/(31/29)	(1) Specific group: — (54.8%) (2) Nonspecific group: — (48.3%)	Only subjects for whom opiates were determined to be the primary drug and who met federal requirements for entry into methadone treatment	AA	Phase I: 5 days per week (2 weeks) Phase II: available on a daily basis (6 months)	Sympathetic Shenmen Kidney Lung Liver	Nonspecific points	(1) Attendance and retention rate (2) Self-reported symptoms (3) Cravings (4) Methadone dose (5) Urinalysis	(1) NS (2) NS (3) NS (4) NS (5) NS	NR

Zhang et al. (2000), [45] China	1	181 (121/60)	(1) Specific group: 27.1 (—) (2) Control group: 25.4 (—)	(1) DSM III for opiate dependence (2) Positive morphine in urine	HANS (The frequency was 2/100 HZ; the intensities were 12–16 mA on arms and 16–26 mA on legs)	Phase I: 4 times per day (3 days) Phase II: Twice a day (3 days) Phase III: Once a day (7 days)	Hegu (LI4) Laogone (PC8) Neiguan (PC6) Waiguan (SJ5) Zusanli (ST36) Sanyinjiao (SP6)	Electrodes were placed at the acupoints without any electrical stimulation	(1) Heart rate (2) Body weight (3) Sleeping time (4) Chilling (5) Pain (6) Anxiety (7) Catarrh (8) Craving	All 8 indices improved significantly (*P* < 0.01)	NR

Montazeri et al. (2002), [46] Iran	2	40 (20/20)	(1) Specific group: 32 (100%) (2) Control group: 31 (100%)	Self-reported with history of heroin or opium addiction less than 6 months	Body acupuncture with manual stimulation	Once per day for 3 days	Hegu (LI4) Neiguan (PC6) Zusanli (ST36) Shenmen (HT7) Taichong (LR3) Dazhui (DU14) Baihui (DU20)	ROD by naloxone	CINA	The acupuncture group had smaller increase in CINA score compared to control group (*P* < 0.05)	No adverse events

Wu et al. (2003), [47] China	1	120 (30/30/30/30)	(1) Acupuncture group: — (—%) (2) Acupuncture plus opium group: — (—%) (3) Opium plus buprenorphine group: — (—%) (4) Opium plus HANS group: — (—%)	(1) CCMD II-R and DSM III-R for: opiate dependence (2) Positive morphine in urine	Body acupuncture with manual stimulation	Phase I: Twice a day (3 days) Phase II: Once a day (7 days)	Sishencong (EX-HN1) Neiguan (PC6) Hegu (LI4) Zusanli (ST36) Sanyinjiao (SP6)	(1) Opium plus buprenorphine therapies (2) Opium plus Han's therapies	(1) Opiate withdrawal scale (2) Craving degree with VAS	(1) Acupuncture group showed significant improvement in withdrawal syndrome after the 6th day (*P* < 0.05) (2) Acupuncture group showed significant improvement in craving degree after the 8th day (*P* < 0.05)	Mild dry mouth

Wen et al. (2005), [48] China	2	220 (111/109)	(1) Acupuncture group: 33.9 (77.5%) (2) Control group: 33.8 (78.0%)	(1) DSM IV for opiate dependence (2) Positive morphine in urine	Body acupuncture with manual stimulation	Once a day (10 days)	Hegu (LI4) Neiguan (PC6) Zusanli (ST36) Waiguan (SJ5) Shenmen (HT7) Sanyinjiao (SP6)	Oral administration of lofexidine hydrochloride	(1) Withdrawal symptom (2) Self-Hamilton anxiety scale	(1) Acupuncture group showed significant improvements in withdrawal syndrome before and after treatment (*P* < 0.05) (2) Acupuncture group showed significant improvement in the score of the self-Hamilton anxiety scale after the 4th day (*P* < 0.001)	NR

Margolin et al. (2005), [49] USA	1	40 (20/20)	(1) Five-needle NADA protocol group: 43.1 (65%) (2) Modified NADA protocol group: 42.6 (55%)	HIV-positive methadone-maintained patients	AA	5 days per week (8 weeks)	Sympathetic Shenmen Kidney Lung Liver	Month 1: Shenmen Month 2: Sympathetic Shenmen Lung	(1) Retention rate (2) Attendance rate (3) Drug use (4) Changes in depression and anxiety	(1) NS (2) NS (3) NS (4) NS	NR

Zeng et al. (2005), [50] China	1	70 (35/35)	(1) Treatment group: 33.1 (83.9%) (2) Control group: 34.2 (80.8%)	(1) DSM III-R for opiate dependence (2) Positive morphine in urine (3) Had opioid withdrawal syndrome	Body acupuncture with manual stimulation	Once a day (10 days)	Baihui (GV20) Dazhui (GV 14) Shendao (GV11) Lingtai (GV10) Zhiyang (GV9) Mingmen (GV4) of the Du channel	Methadone 10-day decrescendo therapy	Scores of daily withdrawal symptoms	Acupuncture group showed significant improvement in withdrawal symptoms on the 1st, 2nd, 4th, 6th, 7th, 8th, 9th, and 10th days (*P* < 0.05 or *P* < 0.01)	NR

Mu et al. (2005), [51] China	2	120 (30/30/30/30)	(1) Acupuncture group I: 29.5 (40%) (2) Acupuncture group II: 29.7 (43.3%) (3) Simulation group: 28.6 (33.3%) (4) Control group: 31.7 (43.3%)	(1) ICD-10 for opiate dependence (2) Negative morphine in urine (3) Had opioid withdrawal syndrome	Body acupuncture with electrical stimulation (The frequency was 5 Hz; the intensity was 5 mA)	3 times a week (10 weeks)	(1) Acupuncture group I: Jiaji (EX-B2) Shenshu (BL23) (2) Acupuncture group II: Neiguan (PC6) Zusanli (ST36) Shenmen (HT7) Sanyinjiao (SP6)	(1) Simulation group: ST36, SP6 without electrical stimulation (2) Control group: no treatment	(1) Withdrawal symptom (2) Hamilton anxiety scale (HAMA) (3) Self-rating depression scale (SDS)	In the treatment of 4, 8, 10 weeks, acupuncture groups I and II showed significantly decreased withdrawal syndrome, HAMA, and SDS (*P* < 0.01)	NR

Bearn et al. (2009), [52] UK	3	83 (48/34)	(1) Acupuncture group: 36.2 (73%) (2) Control group: 35.7 (79%)	DSM IV for opiate dependence	AA	Once a day on weekdays (14 days)	Five points in the ear cartilage ridge area (acupoints not mentioned)	Application of oil to the ear followed by the attachment of 5 metal clips	(1) Withdrawal symptoms (2) Craving	(1) NS (2) NS	NR

*Note*. Code. NR: not reported; NS: not significant; AA: auricular acupuncture; HANS: Han's acupoint nerve stimulator; ROD: rapid opiate detoxification; CINA: clinical institute narcotic assessment; CCMD: Chinese Classification of Mental Disorders; DSM: The Diagnostic and Statistical Manual of Mental Disorders; ICD: International Statistical Classification of Diseases and Related Health Problems; VAS: visual analogue scale.

**Table 2:** Summary of main acupoints/sites selected in the reviewed studies.

Acupoints/sites	Frequency of appearance (*N*)	Percentage (*N*/22 × %)
Zusanli (ST36)	7	31.82
Sanyinjiao (SP6)	6	27.27
Hegu (LI4)	6	27.27
Neiguan (PC6)	5	22.72
Shenmen (HT7)	3	13.64
Laogone (PC8)	3	13.64
Sympathetic (ear)	3	13.64
Shenmen (ear)	3	13.64
Kidney (ear)	3	13.64
Lung (ear)	3	13.64
Liver (ear)	2	9.09
Waiguan (SJ5)	2	9.09
Baihui (GV20/DU20)	2	9.09
Dazhui (GV14/DU14)	2	9.09
Jiaji (EX-B2)	1	4.55
Shenshu (BL23)	1	4.55
Sishencong (EX-HN1)	1	4.55
Taichong (LR3)	1	4.55
Shendao (GV11)	1	4.55
Lingtai (GV10)	1	4.55
Zhiyang (GV9)	1	4.55
Mingmen (GV4)	1	4.55

*Note*. The sum was 22 for the percentage calculation.

**Table 3:** Methodological quality scores.

	Washburn et al. [43]	Wells et al. [44]	Zhang et al. [45]	Montazeri et al. [46]	Wu et al. [47]	Wen et al. [48]	Margolin et al. [49]	Zeng et al. [50]	Mu et al. [51]	Bearn et al. [52]
(1) Was the study described as randomized?	V	V	V	V	V	V	V	V	V	V
(2) Was the randomization scheme described and appropriate?	x	V	x	V	x	V	x	x	V	V
(3) Was the study described as double-blind?	x	x	x	x	x	x	x	x	x	x
(4) Was the method of double-blinding appropriate?	x	x	x	x	x	x	x	x	x	x
(5) Was there a description of dropouts and withdrawals?	x	V	x	x	x	x	x	x	x	V

Results	1	3	1	2	1	2	1	1	2	3

V: yes = 1; x: no = 0; low quality, 0–2; high quality, 3–5.
